# Subcutaneous Sweet’s Syndrome Presenting With a Single Cutaneous Lesion on the Thigh

**DOI:** 10.7759/cureus.67466

**Published:** 2024-08-22

**Authors:** Joohyung Youh, Takuya Mizukami, Yuri Nagata, Kei Ito

**Affiliations:** 1 Department of Dermatology, JR Sapporo Hospital, Sapporo, JPN

**Keywords:** panniculitis, erythema nodosum, neutrophilic dermatoses, sweet’s syndrome, subcutaneous sweet’s syndrome

## Abstract

Sweet’s syndrome (SS), also known as acute febrile neutrophilic dermatosis, manifests as tender, erythematous skin lesions such as papules, nodules, and plaques that may appear vesicular or pustular. The condition is characterized by widespread infiltrates mainly consisting of mature neutrophils, usually in the upper dermis. Erythema nodosum (EN) is a form of septal panniculitis marked by tender, erythematous lesions primarily appearing on the lower legs. Additionally, subcutaneous Sweet’s syndrome (SSS) is a rare variant of SS that mainly involves the subcutaneous adipose tissue. Skin lesions in SSS generally present as tender, erythematous subepidermal nodules on the extremities, morphologically resembling EN. Both EN and SS can present with fever, malaise, gastrointestinal disturbances, lymphadenopathy, arthralgia, increased white blood cell (WBC) count with neutrophilia, elevated C-reactive protein (CRP), and elevated erythrocyte sedimentation rate (ESR), making differentiation between them often challenging. Therefore, histopathologic evaluation is necessary for an accurate diagnosis. In our case, the patient exhibited a very painful plaque measuring 20 cm in diameter on the upper thigh without significant neutrophil infiltration in the dermis, but with subcutaneous septal neutrophil infiltration. Generally, SS shows stronger leukocytosis with neutrophilia than EN does. Considering the clinical symptoms, laboratory results, and clinical progression, the clinicopathological findings aligned more closely with SSS than EN. This article describes a rare case of SSS presenting with a single cutaneous lesion on the thigh, which mimicked the histopathological features of EN.

## Introduction

Acute febrile neutrophilic dermatosis, also known as Sweet’s syndrome (SS), presents with painful, erythematous skin lesions such as papules, nodules, and plaques that can sometimes appear vesicular or pustular. SS is characterized by diffuse infiltrates predominantly composed of neutrophils, usually in the upper dermis. While SS primarily involves the dermis, the neutrophilic infiltration can extend into the subcutaneous tissue, resulting in neutrophilic panniculitis [1,2].

Erythema nodosum (EN), a common type of panniculitis, is the prototypical example of septal panniculitis. Clinically, EN is characterized by the sudden onset of highly tender, non-ulcerating erythematous nodules and plaques, typically measuring between 3 and 6 cm in diameter. These lesions are usually bilateral and symmetrical, predominantly affecting the lower legs. EN typically resolves spontaneously within one to six weeks. It is often accompanied by systemic symptoms such as fever, malaise, headache, gastrointestinal disturbances (including nausea and vomiting), lymphadenopathy, and joint pain, particularly in the ankles and knees [3].

Both EN and SS can be associated with elevated white blood cell (WBC) counts with neutrophilia, increased C-reactive protein (CRP) levels, and a heightened erythrocyte sedimentation rate (ESR), with greater elevation typically seen in SS [4]. There are slight differences between SS and EN, with EN generally showing milder leukocytosis and CRP elevation compared to SS, which often presents with higher rates of fever, leukocytosis, and elevated CRP or ESR. These differences can help differentiate between the two conditions [5,6].

## Case presentation

**Figure 1:**
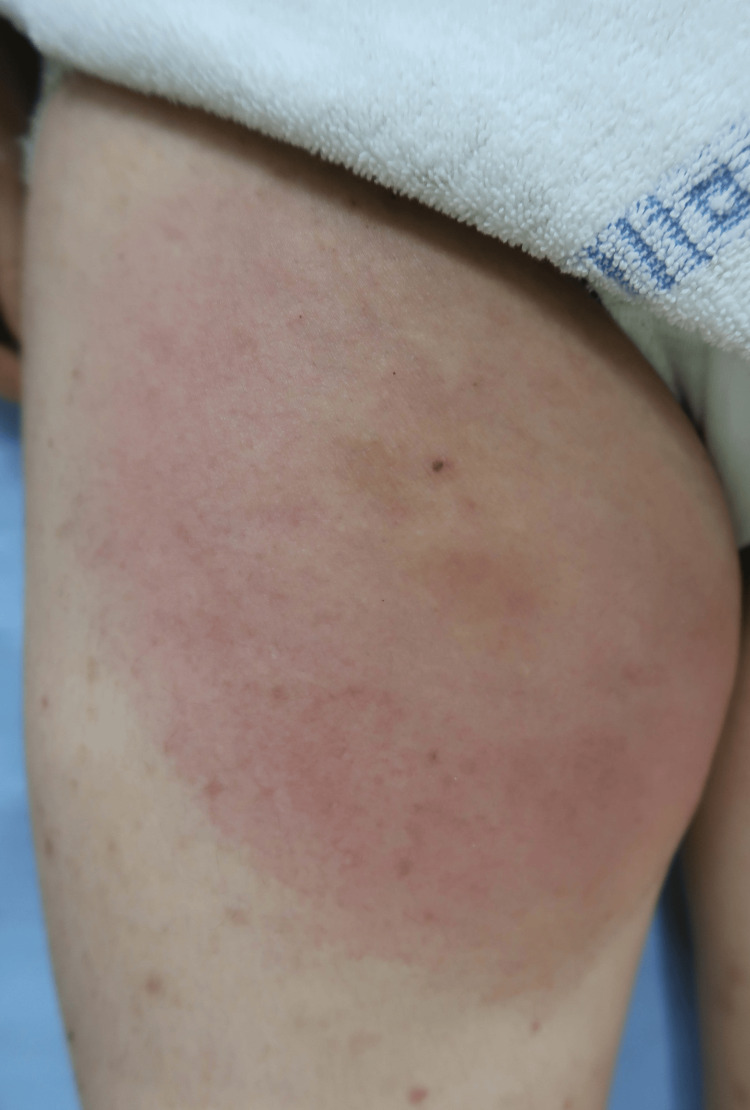
Initial visit An erythematous plaque with extreme tenderness is noted on the patient's right thigh.

A 36-year-old woman with a medical history of only atopic dermatitis presented with an intensely painful cutaneous lesion on her right thigh, accompanied by a high fever reaching 41.4°C that had begun two days prior. She also experienced malaise, headache, nausea, vomiting, and joint pain. The symptoms had a sudden onset. Upon physical examination, an erythematous plaque approximately 20 cm in diameter with extreme tenderness was observed on her right upper thigh (Figure 1).

There were no bullae, erosions, or ulcers present. Given her ongoing treatment for atopic dermatitis, eczema was observed over her entire body, but there had been no recent exacerbation of her condition. No oral or genital cutaneous lesions were detected. She reported no history of infectious diseases, except for a five-day episode of mild upper respiratory infection symptoms, including cough and sore throat, two weeks prior, and she was not taking any medications. She also reported no history of inflammatory bowel disease, hematologic disease, or Behçet's disease. Her obstetric history includes gravidity one and parity one, and she has no notable medical history related to obstetrics and gynecology. Laboratory tests and a CT scan for further evaluation revealed subcutaneous inflammation in the right upper thigh, leukocytosis with a WBC count of 12,900/μL, neutrophilia (92.5%), elevated CRP levels (up to 16.5 mg/dL), and an increased ESR (53 mm/h) (Table 1).

**Table 1:** Laboratory Investigations on Days 1, 2, 3, 4, 7, and 20 of Admission WBC: White blood cell, CRP: C-reactive protein, ESR: Erythrocyte sedimentation rate, sIL-2R: Soluble interleukin 2 receptor

Laboratory Parameters	Day 1	Day 2	Day 3	Day 4	Day 7	Day 20	Reference Range
WBC Count (x10³/μL)	12.9	12.9	15.7	11.0	10.4	6.9	3.5-9.0
Neutrophils (%)	92.5	92.9	91.1	89.2	74.5	62.7	40-70
CRP (mg/dL)	16.5	23.5	19.5	7.9	0.5	0.01	0-0.2
Procalcitonin (ng/dL)	0.18			0.14	<0.02		0-0.05
ESR (mm/hr)	53			14	6		0-20 (in women)
sIL-2R (U/mL)	469			243			121-613

**Figure 2:**
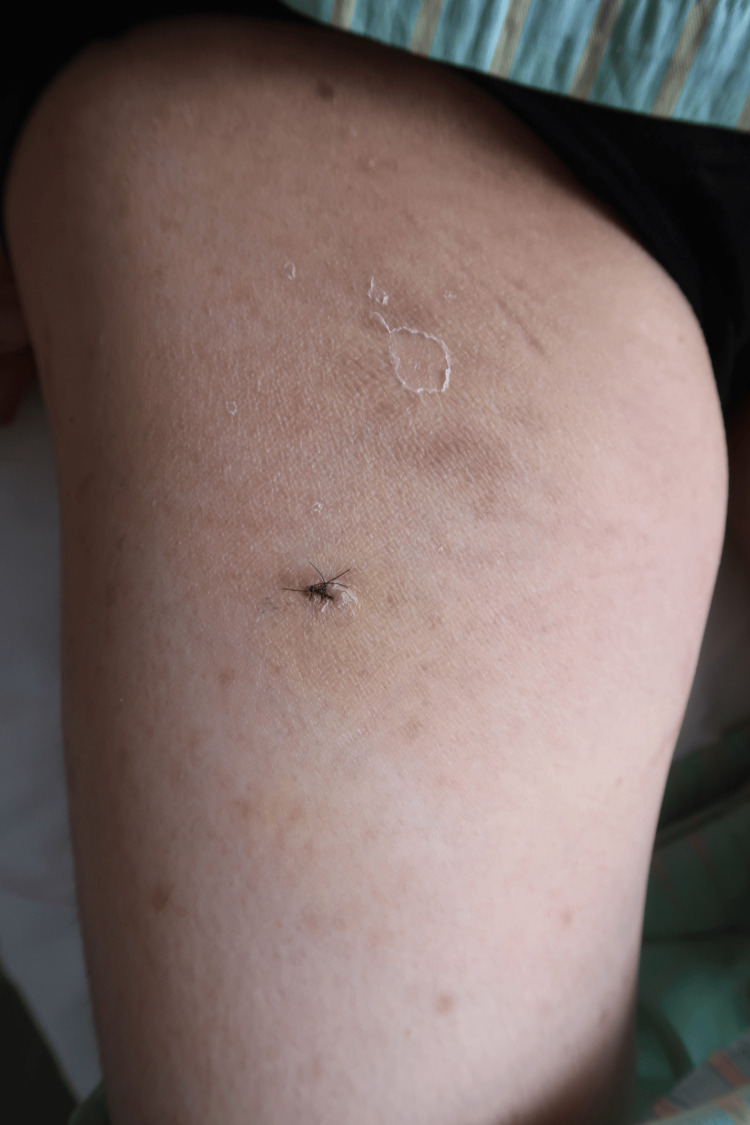
Seven days after administration of corticosteroids The erythematous plaque on the right thigh has begun to fade.

Initially, we began treatment with the broad-spectrum antibiotics tazobactam and piperacillin (TAZ/PIPC), as a infectious panniculitis could not be ruled out, and we used non-steroidal anti-inflammatory drugs (NSAIDs) to reduce the high fever and severe pain. Despite this, follow-up laboratory tests the next day showed increased WBC counts, a higher neutrophil ratio, elevated CRP levels (23.5 mg/dL), and the high fever persisted (Table 1). Based on the clinical progression, laboratory data, and skin lesion morphology, we considered the possibility of atypical presentation of EN and neutrophilic dermatitis, particularly Sweet’s syndrome. Consequently, we initiated treatment with prednisolone at 30 mg/day (0.5 mg/kg/day) along with a five-day course of TAZ/PIPC as a diagnostic measure. Additionally, we included colchicine at 1.5 mg/day to control neutrophil chemotaxis. Following the commencement of prednisolone, the patient’s clinical symptoms, including high fever and severe tenderness, showed significant improvement, and the erythematous plaque on the right thigh began to fade (Figure 2). 

After discontinuing TAZ/PIPC, there was no increase in WBC count or CRP levels during prednisolone therapy. Therefore, we gradually reduced the doses of prednisolone and colchicine, with no noted aggravation of symptoms (Table 1).

**Figure 3:**
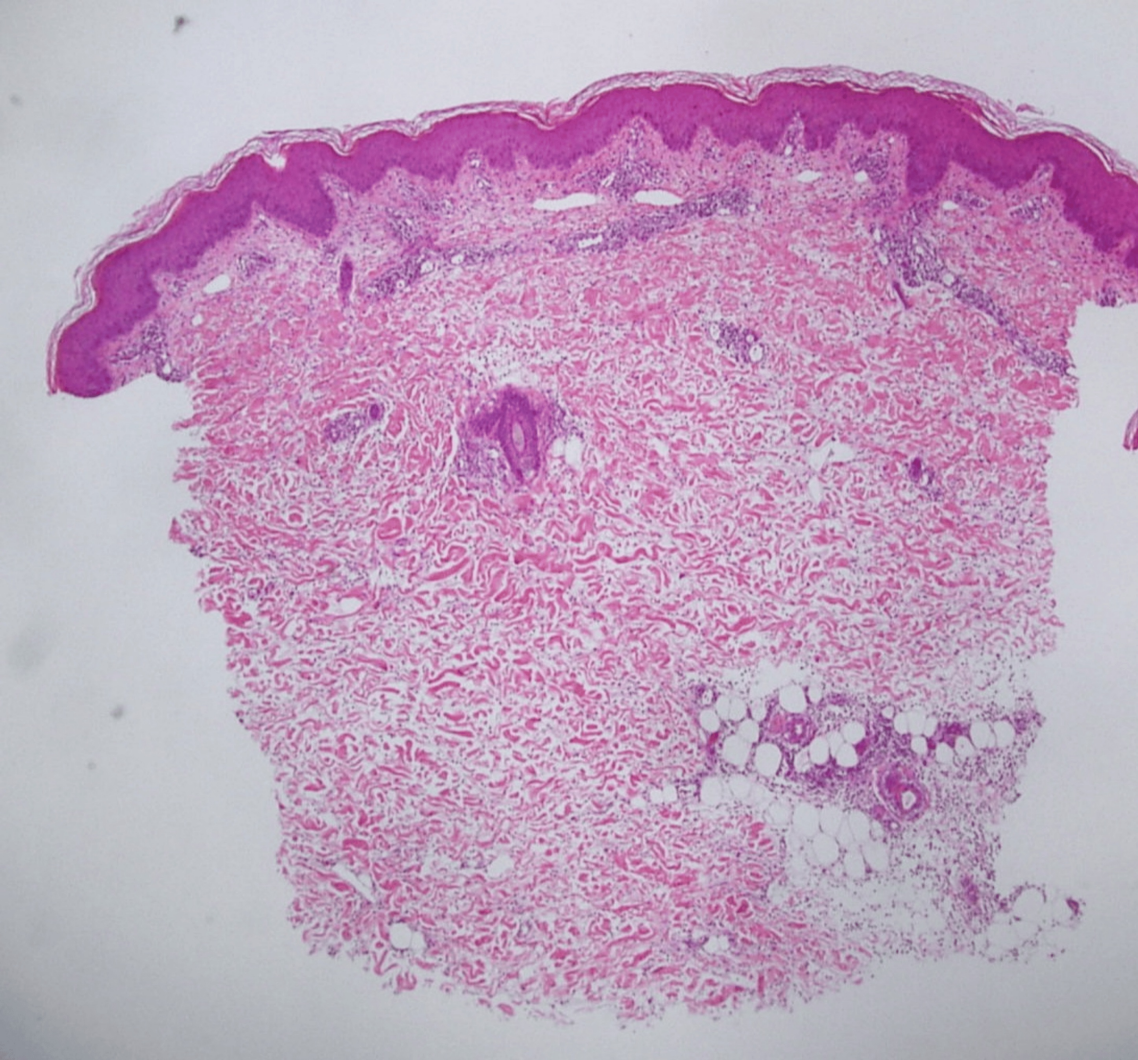
Histopathologic findings (Hematoxylin and eosin, 40x magnification) Subcutaneous septal infiltration of neutrophils, histiocytes, and lymphocytes is observed.

**Figure 4:**
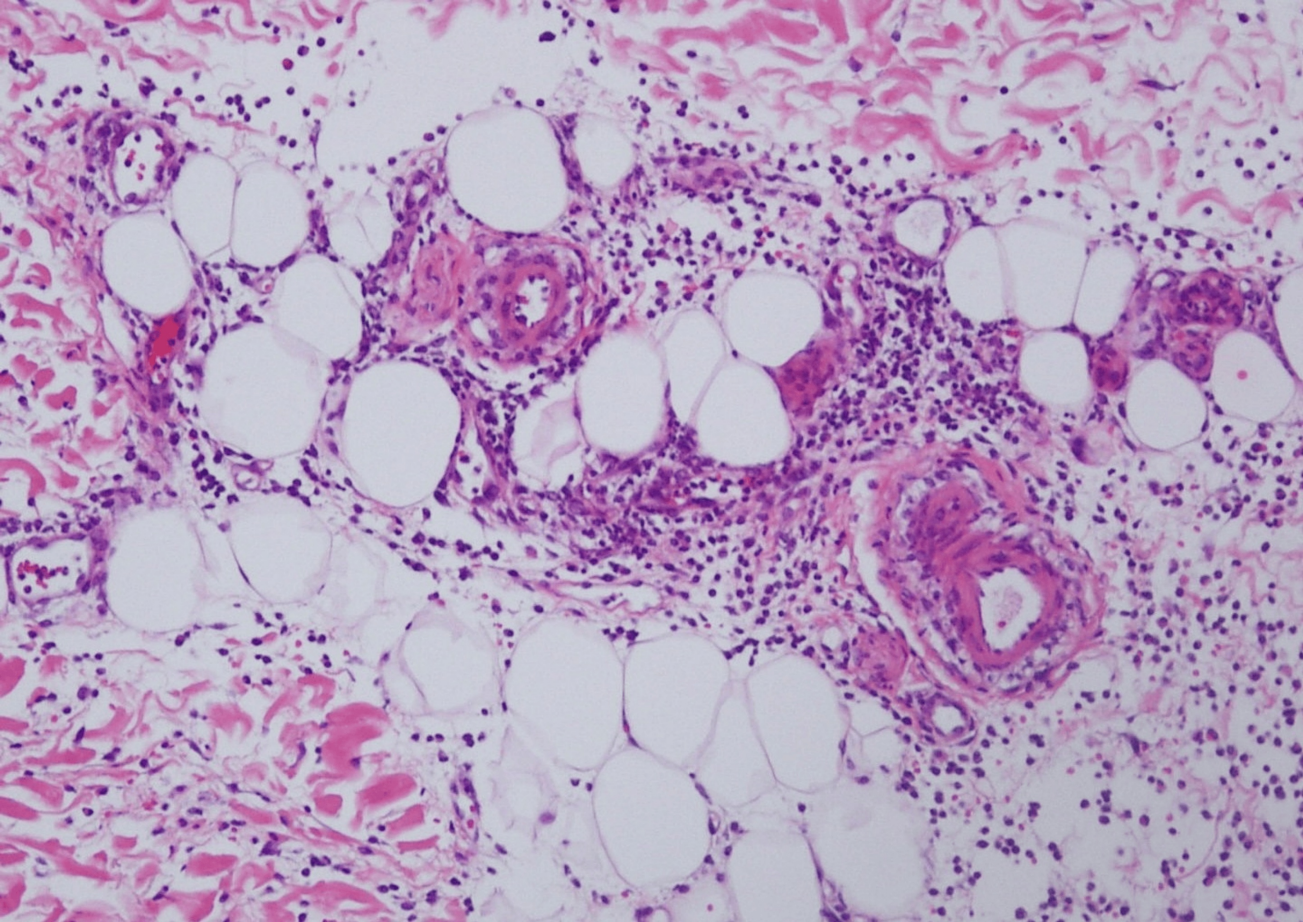
Histopathologic findings (Hematoxylin and eosin, 100x magnification) Subcutaneous septal infiltration of neutrophils, histiocytes, and lymphocytes is observed.

**Figure 5:**
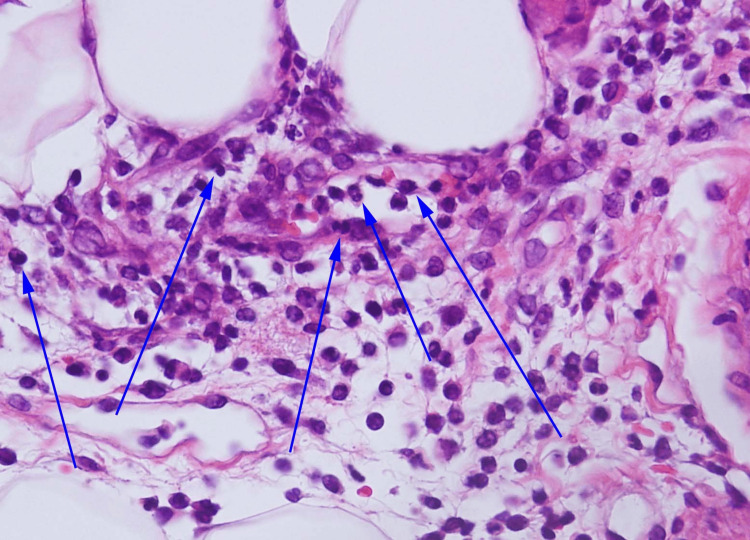
Histopathologic findings (Hematoxylin and eosin, 400x magnification) Subcutaneous septal infiltration of neutrophils (blue arrows), histiocytes, and lymphocytes is observed.

A skin specimen from the painful erythematous plaque on the thigh revealed inflammatory infiltration, including neutrophils, histiocytes and lymphocytes, primarily in the subcutaneous septal area (Figures 3-5). Atypical lymphocytes were not noted. An ultrasound scan of her left shoulder, where she reported arthralgia, did not reveal any definitive signs of arthritis.

## Discussion

Some researchers have identified a subtype of Sweet's syndrome in which the neutrophilic infiltrates are confined to the subcutaneous tissue, referred to as subcutaneous Sweet’s syndrome. In the subcutaneous fat, neutrophilic infiltrates can be present in the lobules, the septa, or both [2-3]. Neutrophilic panniculitis represents a histological pattern found in several conditions, including erythema induratum, early EN, infectious panniculitis, pancreatic panniculitis, Behcet’s disease, Crohn’s-associated neutrophilic panniculitis, and SSS. Accurate diagnosis requires comprehensive clinicopathologic correlation [2,7].

Differentiating SSS from EN is particularly challenging. Reports have indicated that some patients with SS may present with leg nodules that are clinically and histologically similar to EN. In these instances, the neutrophilic infiltrates are located in the septa. While fully developed EN typically manifests as septal panniculitis with lymphocytic and histiocytic involvement, early-stage EN may exhibit mixed infiltrates with lobular involvement, complicating differentiation from other neutrophilic panniculitis [2,3,7,8].

Furthermore, Ginarte and Toribio suggested that EN should be classified as a neutrophilic dermatosis, highlighting four key features to substantiate the link between SS and EN: both are reactive dermatoses triggered by similar stimuli, their clinical manifestations on the legs can be quite similar, the two conditions can occur simultaneously or sequentially in the same patient, and both respond to similar treatments [8]. Given that early-stage EN often displays septal neutrophilic infiltrates, it is reasonable to include EN within the neutrophilic dermatoses [8].

However, there are slight differences between SS and EN. A report of 100 cases of EN indicated that leukocytosis and CRP elevation are generally milder in EN than in SS. Leukocytosis (WBC counts ≥10,000/mm³) is noted in 14% of EN cases, and CRP elevation over 12 mg/dL is observed in only 13% of EN cases [5]. In contrast, an analysis of 44 SS cases by Abbas et al. showed fever in 70%, leukocytosis in 55%, arthralgia in 50%, and high CRP or ESR in 60% of SS patients without a paraneoplastic background [6]. These slight differences may provide useful clues for differentiating between SS and EN.

In our case, although the patient presented with a single cutaneous lesion in an atypical location, the morphology and histopathological characteristics of the lesion on the thigh resembled those of EN rather than classical SS. Additionally, no obvious paraneoplastic background was noted. However, laboratory findings, including significant leukocytosis with elevated neutrophil counts and CRP, along with a dramatic response to corticosteroids, supported the diagnosis of SSS. Given the clinical and histopathological similarities between these two conditions, which are thought to potentially belong to the same disease spectrum, distinguishing between them can be challenging.

## Conclusions

Our report discusses a case of subcutaneous Sweet's syndrome, a rare variant of Sweet's syndrome, particularly notable for presenting with a single cutaneous lesion. This variant is characterized by subcutaneous inflammatory infiltration, significant leukocytosis with markedly elevated neutrophil counts, resistance to antibiotic therapy, and a dramatic response to corticosteroid treatment. To date, there have been few reports of SSS, and to our knowledge, only one other case of SSS with a single cutaneous lesion has been documented. Distinguishing panniculitis with a single cutaneous lesion and severe systemic symptoms, such as high fever, is challenging based solely on clinical and laboratory data. Early intervention with anti-inflammatory treatments, including corticosteroids, is crucial in SSS, as antibiotic therapy is generally ineffective. Dermatologists should consider SSS when encountering panniculitis-like lesions and employ comprehensive approaches, including histopathological evaluation.
